# The effect of nordic walking with poles with an integrated resistance shock absorber on muscle stiffness and elasticity indicators in postmenopausal women

**DOI:** 10.3389/fphys.2025.1587514

**Published:** 2025-05-21

**Authors:** Krystian Wochna, Rafał Stemplewski, Piotr Leszczyński, Piotr Kocur

**Affiliations:** ^1^ Department of Swimming and Water Lifesaving, Poznan University of Physical Education, Poznan, Poland; ^2^ Department of Digital Technologies in Physical Activity, Poznan University of Physical Education, Poznan, Poland; ^3^ Department of Internal Medicine and Metabolic Disorders, Poznan University of Medical Sciences, Poznan, Poland; ^4^ Department of Musculoskeletal Physiotherapy, Poznan University of Physical Education, Poznan, Poland

**Keywords:** muscle stiffness, muscle elasticity, ageing, physical activity, postmenopausal women

## Abstract

**Introduction:**

Understanding the impact of physical activity and its relationship with changes in the biomechanical parameters of the myofascial system at different stages of menopause is important for planning and implementing effective action strategies. The reduction in elasticity and increase in stiffness in upper limb muscles, including in particular tonic muscles, lead to mobility limitations and increased risk of pain. Therefore, the aim of the present study was to evaluate and compare the impact of different types of Nordic walking training on muscle stiffness and elasticity indicators in postmenopausal women.

**Methods:**

Thirty women were randomly assigned to two training groups using different kind of poles and completed 8 weeks of training program. Before starting the training programs and after their completion, total fat, lean body mass were measured, along with muscle stiffness and elasticity indicators. Main calculations was based on 2 × 2 analysis of variance ANOVA with repeated measures.

**Results:**

The significant interaction effect was found in differences between the two groups of women with respect to the upper trapezius muscle (p = 0.037). Significant “time” effects were observed for the triceps brachii and brachioradialis muscles (p = 0.032, p = 0.028, respectively) as a result of increased post-intervention muscle stiffness for all participants.

**Discussion:**

The upper trapezius may be the muscle most strongly engaged during the training, which may lead to sustained passive changes and should have an influence on recommendations on the use of RSA poles in population of postmenopausal women in selected clinical conditions. NW training with RSA poles may be an alternative to traditional NW training for individuals whose physical fitness and health status allows them to engage in such training.

## 1 Introduction

Physical fitness is crucial for the health of perimenopausal women (Sipilä et al., 2020), as some of its parameters related to muscle function and morphology deteriorate in this period of life. Understanding the impact of physical activity and its relationship with changes in the biomechanical parameters of the myofascial system at different stages of menopause is important for planning and implementing effective action strategies. The aim of such strategies is to delay functional decline and development of myofascial problems associated with ageing and the menopausal transition (Macêdo et al., 2023; Walankar et al., 2023).

Previous studies have shown that gender and age are very significant factors in the development of upper body myofascial pain. Women have a higher lifetime prevalence of neck pain compared to men, and the risk of neck disorders increases with age (Côté et al., 2008; Cagnie, et al., 2007). Postmenopausal women have a significantly higher prevalence of musculoskeletal symptoms than premenopausal women, with a peak prevalence during early postmenopause (Gao et al., 2013). This may be due to sex-related changes in muscle and connective tissue (Khadilkar, 2019), which have a direct impact on muscle biomechanical indicators such as stiffness and elasticity. These parameters may play a significant role in the development of musculoskeletal disorders and the overall functionality of the muscular system (Chodock et al., 2020). Other authors have shown that upper extremity muscle elasticity decreases, while upper extremity muscle stiffness increases between the ages of 40 and 80 years in both women and men (Lee et al., 2022). Moreover, it has been demonstrated that with ageing, the stiffness of neck muscles increases by approximately 1.5% per year between the third and ninth decade of life (Kocur et al., 2019). As similar findings have been made for lower limb muscles (Agyapong-Badu et al., 2016), it is believed that both muscle stiffness and muscle elasticity can be stable indicators for assessing musculoskeletal ageing.

The age-related reduction in elasticity and increase in stiffness in upper limb muscles, including in particular tonic muscles, lead to mobility limitations, increased risk of limited overall mobility and pain (Wendt and Waszak, 2024). Increased muscle stiffness results in reduced ability of muscles to absorb energy during movement, which has a negative impact on postural control, motor coordination and the muscles torques of the movement generated. This results in increased risk of pain and reduced movement effectiveness (Ličen et al., 2024).

Training programmes combining endurance, strength and balance exercises may prevent age-related progressive structural changes in muscles (Di Lorito et al., 2021). Regular training, including walking exercises, can help to reduce muscle stiffness and improve muscle elasticity, which has so far been mainly demonstrated for lower body muscles (Yekta et al., 2024). Nordic walking (NW) is one of the most commonly recommended forms of physical activity for ageing individuals which is effective in improving different health-related fitness indicators and can be easily adapted to a person’s physical capabilities (Nikitas et al., 2022; Gomeñuka et al., 2019; 2020). The electrical activity of upper limb muscles during NW was examined by Pellegrini et al. (2015); Pellegrini et al. (2017); Pellegrini et al. (2018). However, there have been no studies examining the impact of this type of physical activity on the biomechanical parameters of selected upper limb muscles that are most strongly engaged during NW. Changes in the biomechanical parameters of certain shoulder girdle and upper limb muscles such as increased stiffness and decreased elasticity can, on the one hand, significantly limit the mobility of the neck and the upper limb girdle and, on the other, indicate that the muscles are strongly engaged during NW. The assessment of the parameters is important in maintaining functional capacity in the context of a number of adaptive changes induced by exercise and ageing that take place in the connective tissue (Purslow, 2020) and the muscle tissue (Lakie and Campbell, 2019). One of the contemporary forms of NW, which provides additional resistance and greater overall exercise intensity, involves the use of poles with an integrated resistance shock absorber (RSA), which is an elastic tape between two fixed elements inside the poles (Marciniak et al., 2020). Previous research has shown that the use of such poles results in stronger engagement of the musculoskeletal system and can be an alternative to traditional NW poles (Marciniak et al., 2021; Wochna et al., 2022). However, NW training with RSA poles has been found to be conducive to asymmetrical muscle work, including in particular asymmetry in the work of the shoulder flexors. Consequently, it is necessary to monitor whether the exercise is being performed correctly, especially in women at risk of musculoskeletal pain and degenerative diseases, which are common in this population. The use of RSA poles may thus potentially have various effects on the stiffness of neck and upper limb muscles.

Therefore, the aim of the present study was to evaluate and compare the impact of different types of NW training, namely, NW training with classic poles and NW training with RSA poles, on muscle stiffness and elasticity indicators in postmenopausal women. We assumed that the impact of NW training with RSA poles would be higher than that of NW training with classic poles, which in the future may have an influence on recommendations on the use of RSA poles in this population in selected clinical conditions.

## 2 Materials and methods

### 2.1 Participants

Subjects were recruited by advertisements in local media and at information events. Initially, 47 women who declared good health status without inflammatory disorders based on medical consultation were qualified to participate in the project. All subjects declared that they did not have a history of participating in professional sports. The subjects were asked not to change their dietary habits for the duration of the project, nor to perform any additional physical activity, except for that carried out in the research project and connected to activities in daily life. Women qualified for the study were randomly assigned to one of two groups (walking with classic poles - NW, and with poles with resistance shock absorber–RSA) with use of Excel software (first step—creation of a column with randomized numbers in range 0–0.99 for each participant; second step—assignment to experimental (RSA) and control (NW) groups in the next column with the following function: if (value <0.5,“RSA”,“NW”)).

**FIGURE 1:**
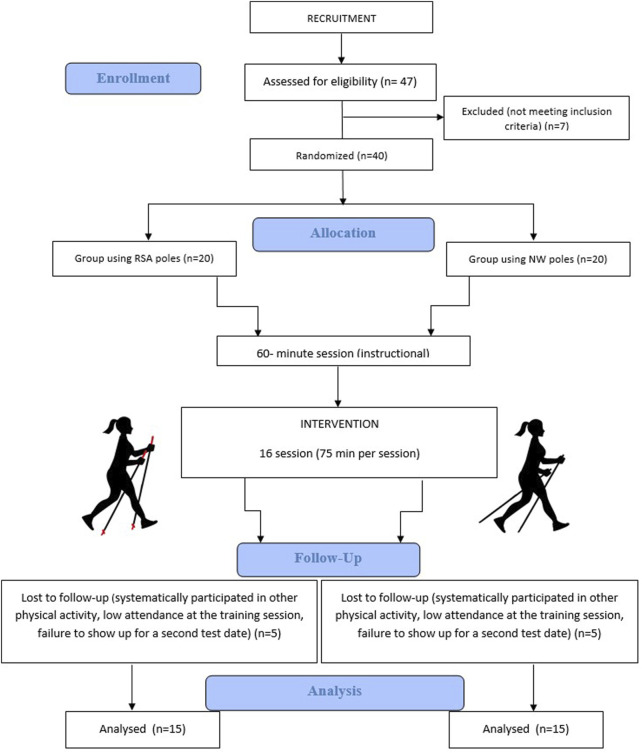
Flow Diagram of the study participants.

Participants who failed to abide by the research protocol and declared that during the study period they had systematically participated in other physical activity classes (n = 10) or had low attendance (less than 80%) at the training sessions (n = 2) or did not appear on the second term of research (n = 5) were excluded from the project. Finally, 30 women (15 RSA; 15 NW) were subjected to statistical analysis (Figure 1). An information sheet was provided to each woman approached to participate in the study and on their agreeing to participate, informed written consent was obtained. All participants provided written informed consent to participate in the study.

### 2.2 Training program

The training program was described based on the study by Marciniak et al. (2020), which forms part of the same research project. The interventions took place twice a week over a period of 8 weeks, comprising 16 training sessions in total. Both groups participated in the sessions simultaneously. Women in the NW group used standard nordic walking poles, while those in the RSA group used poles equipped with integrated resistant shock absorber with an elastic resistance of 4 kg (Slimline Bungy Pump, Sports Progress International AB, Sweden). Each session began with a 10–15-min warm-up. After covering half of the planned distance (approximately 1.7–2.2 km at a pace of about 1 km every 10 min), participants engaged in strength and balance exercises for 15 min. Following the remaining distance, a 15-min stretching session concluded the training. Over the course of the program, the walking distance gradually increased from 3.5 km to 4.5 km, as did the number of exercise repetitions, which rose from eight to 12. The intensity of the exercise was set at 50% of heart rate reserve (HRR) during sessions one to eight, while from sessions 9–16, it was increased to 65%–70% HRR, monitored using heart rate data (HR) (Polar Electro Oy, Kernpele, Finland). Participants were required to attend at least 13 training sessions (80% attendance). The instructor was fully qualified to lead the sessions (International Nordic Walking Association).

### 2.3 Primary outcomes

The primary outcome measures were: muscle stiffness (N/m), defined as the resistance of the myofascial tissue to an external force that has changed its original shape, and muscle elasticity, described as an ability to restore superficial shape and dissipate mechanical energy after the myofascial tissue has been deformed.

#### 2.3.1 Muscle stiffness and elasticity measurements

##### 2.3.1.1 Equipment

Measurements of viscoelastic parameters were taken in the middle of the muscle belly by means of the MyotonPRO device (Myoton AS, Tallin, Estonia).

##### 2.3.1.2 Indicators

In the supine position, we measured stiffness of the descending part of the upper trapezius (UT) muscle, then the long head of the biceps brachii (BB) muscle and brachioradialis muscle (BR) and, finally, the triceps brachii–lateral head (TB).

##### 2.3.1.3 Measurement procedure

Measurement of mechanical muscle parameters was performed twice by the same assessor. The evaluation and measurements took place in the middle of the week between 9 a.m. and noon. The study was conducted 3 days before the start of the training period and 3 days after the end of the training period. Each time, before taking myotonometric measurements, the subject lay in a resting position for approximately 5 min with their legs bent at the knees, upper limbs placed along the torso with 45° elbow flexion and neutral forearm.

##### 2.3.1.4 Reliability

Superficial muscles were selected for the experiment, as they are more strongly engaged during walking due to the technique and gait pattern with poles (Pellegrini et al., 2015). The intra-rater and inter-rater reliability across all of muscles examined in our project indicate that these structures can be assessed reliably (Lettner et al., 2024). During the measurement with MyotonPRO, the midpoint on the long axis was marked for BB and TB (Hermens et al., 2000). The measurement point for the BR was marked 1 cm underneath the elbow flexion line, which was marked between the epicondyles of the humerus, while for the UT, it was marked halfway between the most lateral part of the acromion process and the spinous process of the C7 vertebra (Kocur et al., 2019). The measurement was performed twice for each muscle tested, on both sides of the body. Our statistical analysis, which was performed before combining the results, showed there was no difference between the biomechanical parameters on the left and right side of the body. Therefore, the values of the analysed parameters obtained in successive measurements were first averaged for each side of the body and then again for both sides.

### 2.4 Secondary outcomes

The secondary outcome measures were: segmental total fat and lean body mass.

#### 2.4.1 Fat measurements

Total fat, lean body mass, android and gynoid fat tissue were determined using a Lunar Prodigy Advance densitometer (General Electric, USA). All scans were taken by the same technician, using the same device, which was calibrated daily. Scan analyses were performed using integrated software, according to the manufacturer’s recommendations.

### 2.5 Statistical analysis

Calculations were performed using parametric statistics, based on the assumption of homogeneity of variance between groups, as checked by Levene’s test. Only in the case of arm fat content at pretest was this condition violated. In addition, skewness and kurtosis were checked for the variable. The obtained values of skewness for both groups and kurtosis for the RSA group were within appropriate ranges proposed by George and Mallery (2021): <−1.5; 1.5> and <−2; 2>, respectively. Only the kurtosis value for the NW group was slightly higher (2.30). A full analysis of Levene’s test for all variables, as well as the skewness and kurtosis for arm fat content has been presented in the Supplementary Material. Given that the variable was secondary and the violation of conditions was quite low, the parametric test was also used in this case to keep the analysis homogeneous and also not to lose information about the interaction effect.

All variables were presented with basic statistics, such as mean values, standard deviations and 95% confidence intervals of the mean. To examine any potential differences between groups before the intervention in baseline characteristics, the Student’s t-test was used. To assess the variability of dependent variables, both primary and secondary, we used ANOVA (F-test) with the within-group repeated measures factor “time” (two levels: before and after) and the between-group factor “group” (two levels: NW and RSA). The interaction effects (“group” × “time”) and main effects of “time” and “groups” were estimated, as well as the corresponding effect sizes (partial eta-square). The effect size indicates the percentage of variance explained by each effect of the dependent variable. For detailed *post hoc* comparisons (both pre-post values within groups and between groups in the pre and post conditions), Bonferroni correction was applied. The minimum level of statistical significance was defined as p ≤ 0.05. The study was conducted using Statistica v. 13.0 software (TIBCO Software Inc., Palo Alto, CA, USA).

## 3 Results

The mean age of all participants was 67.0 ± 3.69 years (range 60–55 years). A comparison of baseline characteristics between the study groups before the experiment is shown in Table 1. There were no statistically significant differences between NW and RSA participants.

**TABLE 1:** Mean values, standard deviations, confidence intervals for means, minimum and maximum values for basic characteristics in study groups and the analysis of differences between groups.

	Group NW (n = 15)		Group RSA (n = 15)		t-test (*p* value)
	$\bar{x}\pm \text{SD}$ [mean CI95%]	min - max	$\bar{x}\pm \text{SD}$ [mean CI95%]	min - max	
Age [years]	65.8 ± 3.49 [63.9–67.7]	60–72	68.2 ± 3.59 [66.2–70.2]	62–75	−1.86 (0.074)
Height [m]	1.61 ± 0.06 [1.58–1.65]	1.53–1.73	1.61 ± 0.04 [1.59–1.63]	1.55–1.68	−0.05 (0.957)
Weight [kg]	66.9 ± 10.52 [61.0–72.7]	52–96.5	73.2 ± 9.52 [67.9–78.5]	55.6–92.7	−1.73 (0.095)
BMI [kg/m2]	25.7 ± 3.42 [23.8–27.6]	20–33.3	28.1 ± 3.58 [26.1–30.1]	22.3–34.0	−1.88 (0.070)
Arms fat [%]	37.8 ± 6.56 [35.9–39.8]	29.1–44.3	38.4 ± 5.79 [34.7–42.0]	25.2–47.2	−0.28 (0.779)
Trunk fat [%]	42.1 ± 3.48 [38.9–45.2]	31.8–50.7	44.5 ± 6.59 [41.1–47.9]	32.3–55.4	−1.13 (0.267)

$\bar{x}$ – mean value, SD–standard deviation, CI–confidence intervals, BMI–body mass index, NW–Nordic walking, RSA–resisnce shock absorber.

**FIGURE 2:**
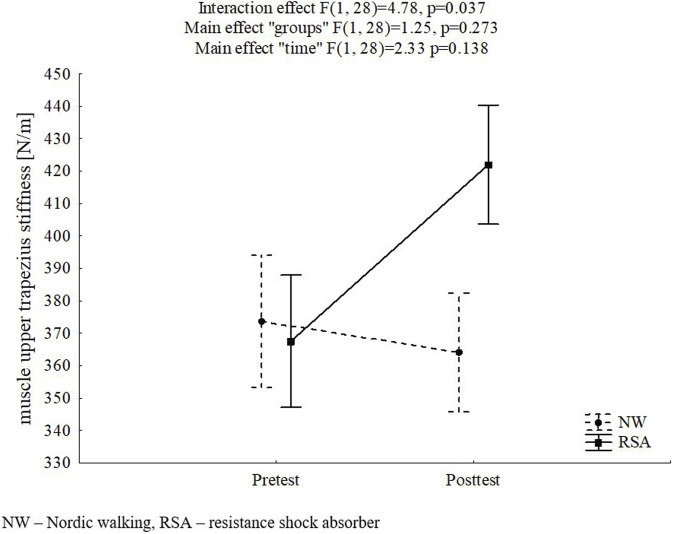
Mean and standard error of measurement values of muscle upper trapezius stiffness in pre- and post-test for study groups, and results of two-way ANOVA with repeated measures.

The results of the main analysis of the primary outcomes are shown in Table 2. For muscle stiffness, a “time” x “group” interaction effect was found for UT (F = 4.78, p = 0.037, η^2^ = 0.146). This result was associated with increased post-test values in the BB group, but the differences (within the group as well as post-test between groups) were not statistically significant–Figure 2.

**TABLE 2:** Mean values, standard deviations, confidence intervals for means for BMI and percentage fat content at arms and trunk in pre- and post-test measurements in study groups, and the results of analysis of two-way ANOVA with repeated measurements.

	Pretest		Posttest		“Groups” F (p value) η^2^	“Time” F (p value) η^2^	“Groups” x “time” F (p value) η^2^
	Group NW	Group RSA	Group NW	Group RSA			
	$\bar{x}\pm \text{SD}$ [mean CI95%]	$\bar{x}\pm \text{SD}$ [mean CI95%]	$\bar{x}\pm \text{SD}$ [mean CI95%]	$\bar{x}\pm \text{SD}$ [mean CI95%]			
*Muscle stiffness*							
UT [N/m]	373.7 ± 63.1 [338.8–408.7]	367.6 ± 92.1 [316.5–418.6]	364.0 ± 53.9 [334.2–393.9]	422.0 ± 84.7 [375.1–468.9]	1.25 (0.273) 0.043	2.33 (0.138) 0.077	**4.78 (0.037)** **0.146**
BB [N/m]	245.8 ± 16.6 [236.6–255.0]	238.9 ± 18.0 [228.9–248.8]	242.4 ± 23.3 [229.4–255.3]	251.2 ± 38.8 [229.7–272.7]	0.02 (0.891) 0.001	0.47 (0.499) 0.017	1.47 (0.235) 0.050
TB [N/m]	248.4 ± 23.2 [235.5–261.2]	227.5 ± 29.7 [211.0–243.9]	256.8 ± 48.8 [229.8–283.8]	256.0 ± 36.7 [235.6–276.3]	1.13 (0.296) 0.039	**5.10 (0.032)** **0.154**	1.49 (0.232) 0.051
BR [N/m]	249.8 ± 41.7 [226.7–272.9]	252.5 ± 31.8 [234.8–270.1]	279.0 ± 44.8 [254.2–303.7]	268.7 ± 31.9 [251.1–286.4]	0.15 (0.704) 0.005	**5.38 (0.028) 0.161**	0.44 (0.515) 0.015
*Muscle elasticity*							
UT [Log.dec.]	1.40 ± 0.18 [1.30–1.50]	1.45 ± 0.26 [1.30–1.59]	1.50 ± 0.14 [1.42–1.57]	1.45 ± 0.19 [1.35–1.56]	0.00 (0.967) 0.000	1.12 (0.299) 0.038	0.90 (0.351) 0.031
BB [Log.dec.]	2.07 ± 0.37 [1.86–2.27]	1.83 ± 0.45 [1.58–2.08]	1.79 ± 0.42 [1.56–2.02]	1.95 ± 0.56 [1.64–2.26]	0.10 (0.750) 0.004	0.44 (0.513) 0.015	2.56 (0.121) 0.084
TB [Log.dec.]	2.25 ± 0.40 [2.023–2.47]	1.94 ± 0.58 [1.62–2.26]	1.76 ± 0.44 [1.51–2.0]	1.90 ± 0.62 [1.55–2.24]	0.48 (0.493) 0.017	3.30 (0.080) 0.105	2.34 (0.137) 0.077
BR [Log.dec.]	1.26 ± 0.13 [1.18–1.33]	1.36 ± 0.18 [1.26–1.46]	1.25 ± 0.21 [1.14–1.37]	1.29 ± 0.14 [1.21–1.37]	2.83 (0.104) 0.092	0.82 (0.373) 0.028	0.51 (0.483) 0.018

$\bar{x}$ , mean value, SD, standard deviation, CI, confidence intervals; F, test value in analysis of variance ANOVA; η^2^, partial eta squared; UT, muscle upper trapezius; BB, muscle biceps brachi–long head; TB, muscle triceps brachi–lateral head; BR, muscle brachioradialis; NW, Nordic walking; RSA, resistance shock absorber.

In addition, statistically significant “time” effects were observed for TB and BR muscles (F = 5.1, p = 0.032 η^2^ = 0.154 and F = 5.38, p = 0.028, η^2^ = 0.161, respectively) as a result of increased post-intervention muscle stiffness for all participants.

No statistically significant interaction effects or main effects were found for muscle elasticity.

Analysis of secondary outcomes (Table 3) revealed a statistically significant “time” effect for trunk fat content (F = 4.23, p = 0.049, η^2^ = 0.131), which was associated with a reduction in the index value after the intervention–detailed within-group comparisons were not significant.

**TABLE 3:** Mean values, standard deviations, confidence intervals for means for BMI and percentage fat content at arms and trunk in pre- and post-test measurements in study groups, and the results of analysis of two-way ANOVA with repeated measurements.

	Pretest		Posttest		“Groups” F (p value) η^2^	“Time” F (p value) η^2^	“Groups” x “time” F (p value) η^2^
	Group NW	Group RSA	Group NW	Group RSA			
	$\bar{x}\pm \text{SD}$ [mean CI95%]	$\bar{x}\pm \text{SD}$ [mean CI95%]	$\bar{x}\pm \text{SD}$ [mean CI95%]	$\bar{x}\pm \text{SD}$ [mean CI95%]			
BMI [kg/m2]	25.7 ± 3.42 [23.8–27.6]	28.1 ± 3.58 [26.1–30.1]	26.1 ± 3.39 [24.2–27.9]	28.8 ± 4.25 [26.4–31.1]	3.87 (0.059) 0.12	2.27 (0.143) 0.07	0.20 (0.657) 0.007
Arms fat [%]	37.8 ± 6.56 [35.9–39.8]	38.4 ± 5.79 [34.7–42.0]	37.5 ± 4.08 [35.3–39.8]	38.0 ± 6.52 [34.4–41.6]	0.07 (0.792) 0.003	0.88 (0.358) 0.030	0.01 (0.921) 0.000
Trunk fat [%]	42.1 ± 3.48 [38.9–45.2]	44.5 ± 6.59 [41.1–47.9]	41.6 ± 6.07 [38.2–45.0]	43.6 ± 6.58 [39.9–47.2]	1.01 (0.325) 0.035	**4.23 (0.049)** **0.131**	0.49 (0.490) 0.017

$\bar{x}$ , mean value; SD, standard deviation; CI, confidence intervals; F, test value in analysis of variance ANOVA; η^2^, partial eta squared; BMI, body mass index; NW, Nordic walking; RSA, resistance shock absorber.

## 4 Discussion

The present study analysed the impact of 8-week NW training programmes with RSA poles and classic poles on the biomechanical indicators of selected upper limb and shoulder girdle muscles. The most important finding from the study was a statistically significant increase in the stiffness of the UT in RSA participants, with no increase observed in NW participants. The study showed that the myotonometry-measured stiffness of the TB and BR muscles increased, whereas the stiffness of the BB muscle remained unchanged in both RSA participants and NW participants. Thus, the use of a resistance shock absorber did not have an influence on the stiffness of TB, BR and BB muscles. In addition, the change in trunk and shoulder fat content in NW participants was similar to that observed in RSA participants. Previous research by other authors has shown that cutaneous and subcutaneous tissue thickness may have an influence on muscle biomechanical indicators (Grześkowiak et al., 2023; Mencel et al., 2021). Our study showed that while there was a significant change in trunk fat content in the participants included in the study, this did not have a significant influence on the stiffness of the UT as the change was similar for both the RSA and NW groups.

The role of the active biomechanical indicators of muscles in the transfer of muscle tension has been studied by other authors (Mukund and Subramaniam, 2020). However, further research is necessary on the impact of exercise on muscle biomechanical indicators in order to better understand the relationship between the parameters and the quality and intensity of dynamic and static load. Previous studies have reported increased muscle stiffness as a positive effect of training requiring explosive and dynamic movements for the flexors and extensors of the knee and the Achilles tendon (Fouré et al., 2012; Kyung et al., 2024). At the same time, it is worth noting that muscle stiffness decreases or remains unchanged in response to selected forms of training or even long-distance running in the first minutes after exercise (Maćkała et al., 2023; Rotllan et al., 2024), but may increase again and even exceed pre-training stiffness values a few days or several hours after training (Rey et al., 2024). This is consistent with the observation we made several dozen hours post-training. Importantly, changes in passive muscle properties may depend on location in the muscle and intensity of training. In their study, Kawczyński et al. (2018) examined the impact of eccentric exercise on the UT. The authors found changes in the biomechanical properties of the muscle 24 h post-training - stiffness for musculotendinous sites increased, while stiffness for the muscle belly decreased. The finding is consistent with findings from a study by Heredia-Rizo et al. (2020), who found that eccentric training reduces the stiffness of the UT in individuals with neck-shoulder pain.

The different effects of the two training programmes are probably due to the fact that NW training with RSA poles involves the use of a different walking technique and longer poles. Our findings and findings from previous research on the use of RSA poles (Wochna et al., 2022) indicate that great caution should be exercised in recommending regular NW training with RSA poles to people with neck muscle pain and tension-type headaches and individuals with orthopaedic problems with their shoulders. The increase in the stiffness of the UT observed in the present study may thus be unfavourable. In some cases, increased muscle stiffness can limit the mobility of joints and result in neck problems (Taş et al., 2018). The changes in the stiffness of the UT observed after a few weeks of training may be due to sustained transformations in the muscle fibres and fascia in response to slightly more intensive repeated exercise that puts strain on the neck. This may lead to the structural adaptation of the myofascial tissue similar to that caused by sitting, which is associated with an increase in the number of cross-bridges or the cross-linking of collagen fibres as a result of repeated movements (Nishikawa et al., 2018).

Therefore, future research should identify the potential physiological mechanisms underlying changes induced by dynamic and static load. Different forms of training, their volume and intensity shape the passive biomechanical properties of muscles not only immediately after training but especially over a longer period. Previous findings on the subject are not conclusive (Kawama et al., 2024; Kyung et al., 2024). Scientific research clearly indicates that changes in muscle morphology parameters are most significant when muscles are trained at least twice a week (Schoenfeld et al., 2016). Due to the varying physical fitness levels of our participants, we therefore opted for two training sessions per week over a 8-week period. However, it remains unclear how increasing the number of training sessions would affect biomechanical properties, which should also be considered in future studies. Moreover, in certain clinical situations, the changes may be either beneficial or unfavourable. On the one hand, increased UT stiffness is an objective indicator and can be a lasting change in individuals with neck and shoulder pain (Ishikawa et al., 2017). On the other hand, increased muscle stiffness or reduced muscle elasticity may also be an important indicator of changes in muscle tissue enabling a more effective postural response to external forces, guaranteeing an appropriate response to changes in body position, and increase the effectiveness of proprioception (Lakie and Campbell, 2019). Accordingly, it should be stated that future research should assess changes in muscle biomechanical properties in relation to perceived pain threshold (PPT) and range of motion measurements. As well as, includes validated clinical tests of balance and physical function in women.

## 5 Study limitations

The study provides important information on the impact of regular exercise on the biomechanical indicators of muscles. However, it has certain limitations, first of all, the study lacks a control group, which could have strengthened the design and added comparative value. Moreover, the limitation is the small sample size, which should be taken into account when interpreting its findings and planning further research. Moreover, it is impossible to determine whether the changes had an influence on functional capacity, range of motion and development of delayed onset muscle soreness. However, since there have been no previous studies investigating training-induced changes in muscle biomechanical parameters in postmenopausal women, the present study attempted to determine whether such changes occur at all in response to different forms of NW training.

## 6 Conclusion

The 8-week NW training programme with RSA poles resulted in an increase in the stiffness of the UT muscle in RSA participants, with no increase observed in NW participants. This suggests that the UT may be the muscle most strongly engaged during this type of training, which may lead to sustained passive changes. In both NW participants and RSA participants, the stiffness of the TB and BR muscles increased from baseline to post-intervention, whereas the stiffness of the BB remained unchanged. Thus, the use of a resistance shock absorber did not have a significant impact on the stiffness of the TB, BR and BB muscles. NW training with RSA poles may be an alternative to traditional NW training for individuals whose physical fitness and health status allows them to engage in such training.

## Data Availability

The raw data supporting the conclusions of this article will be made available by the authors, without undue reservation.
