# Supplementary material for: The effect of nordic walking with poles with an integrated resistance shock absorber on muscle stiffness and elasticity indicators in postmenopausal women
Source: Front Physiol. 2025 May 21;16:1587514. doi: 10.3389/fphys.2025.1587514 (PMC12133517; doi:10.3389/fphys.2025.1587514)
Supplement: Supplementary file 1 [file DataSheet1.pdf]

Table S1. Results of Levene's test for basic characteristics, primary outcomes, and secondary outcomes at pretest and posttest

	Pretest		Posttest	
	F(1,28)	p	F(1,28)	p
Basic characteristics				
Age [years]	0.15	0.704	-	-
Height [m]	3.80	0.061	3.80	0.061
Weight [kg]	0.02	0.897	0.00	0.948
BMI [kg/m^2]	0.36	0.552	0.90	0.352
Primary outcomes – muscle stiffness				
UT [N/m]	2.15	0.154	3.50	0.072
BB [N/m]	0.00	0.951	0.66	0.423
TB [N/m]	0.72	0.403	0.01	0.931
BR [N/m]	0.33	0.568	1.57	0.220
Primary outcomes – muscle elasticity				
UT [Log.dec.]	1.78	0.193	3.13	0.088
BB [Log.dec.]	0.78	0.386	1.64	0.210
TB [Log.dec.]	1.44	0.240	4.12	0.052
BR [Log.dec.]	0.62	0.439	0.41	0.527
Secondary outcomes				
Arms fat [%]	7.14	0.012	2.87	0.102
Trunk fat [%]	0.11	0.748	0.26	0.615

Table S2. Results of skewness and kurtosis for arm fat content at pretest

	Group NW		Group RSA	
	skewness	kurtosis	skewness	kurtosis
Arms fat [%]	-0,76	2,30	-0,51	-0,74
